# Supplementary material for: Spatial and functional arrangement of Ebola virus polymerase inside phase-separated viral factories
Source: Nat Commun. 2023 Jul 13;14:4159. doi: 10.1038/s41467-023-39821-7 (PMC10345124; doi:10.1038/s41467-023-39821-7)
Supplement: Supplementary file 3 — Description of additional supplementary files [file 41467_2023_39821_MOESM3_ESM.pdf]

# Description of additional supplementary files

**Supplementary Movie 1**: An animated movie sequentially showing tomographic slices and 3D segmentation for the electron tomogram of a subcellular volume, which contains cellular organelles, EBOV viral factories, and sAPEX2-tagged EBOV polymerase. (Related to Figure 6 a, 6b).
