# Supplementary material for: Spatial and functional arrangement of Ebola virus polymerase inside phase-separated viral factories
Source: Nat Commun. 2023 Jul 13;14:4159. doi: 10.1038/s41467-023-39821-7 (PMC10345124; doi:10.1038/s41467-023-39821-7)
Supplement: Supplementary file 5 — Reporting Summary [file 41467_2023_39821_MOESM5_ESM.pdf]

nature portfolio

Corresponding author(s): Erica Ollmann Saphire, PhD

Last updated by author(s): Jun 18, 2023

Reporting Summary

Nature Portfolio wishes to improve the reproducibility of the work that we publish. This form provides structure for consistency and transparency in reporting. For further information on Nature Portfolio policies, see our Editorial Policies and the Editorial Policy Checklist.

Statistics

For all statistical analyses, confirm that the following items are present in the figure legend, table legend, main text, or Methods section.

n/a	Confirmed	
☐	☒	The exact sample size (*n*) for each experimental group/condition, given as a discrete number and unit of measurement
☐	☒	A statement on whether measurements were taken from distinct samples or whether the same sample was measured repeatedly
☐	☒	The statistical test(s) used AND whether they are one- or two-sided<br>*Only common tests should be described solely by name; describe more complex techniques in the Methods section.*
☒	☐	A description of all covariates tested
☐	☒	A description of any assumptions or corrections, such as tests of normality and adjustment for multiple comparisons
☐	☒	A full description of the statistical parameters including central tendency (e.g. means) or other basic estimates (e.g. regression coefficient) AND variation (e.g. standard deviation) or associated estimates of uncertainty (e.g. confidence intervals)
☐	☒	For null hypothesis testing, the test statistic (e.g. *F*, *t*, *r*) with confidence intervals, effect sizes, degrees of freedom and *P* value noted<br>*Give P values as exact values whenever suitable.*
☒	☐	For Bayesian analysis, information on the choice of priors and Markov chain Monte Carlo settings
☒	☐	For hierarchical and complex designs, identification of the appropriate level for tests and full reporting of outcomes
☒	☐	Estimates of effect sizes (e.g. Cohen's *d*, Pearson's *r*), indicating how they were calculated

Our web collection on statistics for biologists contains articles on many of the points above.

Software and code

Policy information about availability of computer code

Data collection	Light microscopy data were collected using ZEISS Zen Black. Electron microscopy data were collected using SerialEM 3.6.12. Luciferase assay data were collected using the Tecan SparkControl Method Editor. Western blot data were collected using the BioRad ImageLab.
Data analysis	Light microscopy data were processed using ZEISS Zen Black. FRAP data were analyzed using Fiji, Schindelin, J. et al. Fiji: an open-source platform for biological-image analysis. Nat Methods 9, 676-682, doi:10.1038/nmeth.2019 (2012). Thin-section electron microscopy data were analyzed using IMOD, Kremer J.R., Mastronarde, D.N., and McIntosh, J.R. (1996) Computer visualization of three-dimensional image data using IMOD. J. Struct. Biol. 116:71-76. 3D tomograms were reconstructed using homegrown softwares as described in Phan, S. et al. 3D reconstruction of biological structures: automated procedures for alignment and reconstruction of multiple tilt series in electron tomography. Adv Struct Chem Imaging 2, 8, doi:10.1186/s40679-016-0021-2 (2017). Tomogram annotations were performed using Thermo Scientific Amira 3.1. Plots were generated with statistical analysis performed all in GraphPad Prism 8.

For manuscripts utilizing custom algorithms or software that are central to the research but not yet described in published literature, software must be made available to editors and reviewers. We strongly encourage code deposition in a community repository (e.g. GitHub). See the Nature Portfolio guidelines for submitting code & software for further information.

Data

Policy information about availability of data

All manuscripts must include a data availability statement. This statement should provide the following information, where applicable:

- Accession codes, unique identifiers, or web links for publicly available datasets
- A description of any restrictions on data availability
- For clinical datasets or third party data, please ensure that the statement adheres to our policy

The authors declare that all other data supporting the findings of this study are available within the article, its Supplementary information files and the source data file.

Research involving human participants, their data, or biological material

Policy information about studies with human participants or human data. See also policy information about sex, gender (identity/presentation), and sexual orientation and race, ethnicity and racism.

Reporting on sex and gender	N/A
Reporting on race, ethnicity, or other socially relevant groupings	N/A
Population characteristics	N/A
Recruitment	N/A
Ethics oversight	N/A

Note that full information on the approval of the study protocol must also be provided in the manuscript.

Field-specific reporting

Please select the one below that is the best fit for your research. If you are not sure, read the appropriate sections before making your selection.

☒ Life sciences ☐ Behavioural & social sciences ☐ Ecological, evolutionary & environmental sciences

For a reference copy of the document with all sections, see nature.com/documents/nr-reporting-summary-flat.pdf

Life sciences study design

All studies must disclose on these points even when the disclosure is negative.

Sample size	No sample-size calculation was performed. Sample-sizes were chosen to enable corresponding statistical analyses, which are specified in each figure legend. For experiments with quantitative analysis, we attempted to have a minimum of two biological replicates with technical triplicates for sufficient reproducibility. For light microscopy-based experiments, we analyzed > 50 cells from at least two biological replicates to ensure representative sampling and to account for biological variability. For electron microscopy-based experiments, we performed at least two biological replicates to ensure reproducibility.
Data exclusions	The FRAP data of VP35-alone condensates were excluded from curve-fitting analysis due to the insufficient number of time-lapse videos acquired in the FRAP experiment. Since the VP35-alone condensates have multiple dark internal regions that lacked fluorescence, only a few of these condensates contain desirable regions for FRAP data acquisition using 1 µm-diameter center spot for photobleaching. With acquired FRAP data, the fluorescence recovery in the VP35-alone condensate was not yet plateaued at the end of time-lapse video, which prevents proper curve-fitting with acquired data points.
Replication	Cell-based assay, including microscopy analyses were repeated in at least two biological replicates with multiple technical replicates specified in the manuscript. For microscopy experiments, multiple fields of view were analyzed in each replicate to ensure representativity and reproducibility. All data were consistent in multiple replicates.
Randomization	Samples were allocated randomly for imaging and analyses.
Blinding	Blinding is not applicable to this study since comparative analyses have either quantitative or phenotypic readouts.

Reporting for specific materials, systems and methods

We require information from authors about some types of materials, experimental systems and methods used in many studies. Here, indicate whether each material, system or method listed is relevant to your study. If you are not sure if a list item applies to your research, read the appropriate section before selecting a response.

Materials & experimental systems

n/a	Involved in the study
☐	☒ Antibodies
☐	☒ Eukaryotic cell lines
☒	☐ Palaeontology and archaeology
☒	☐ Animals and other organisms
☒	☐ Clinical data
☒	☐ Dual use research of concern
☒	☐ Plants

Methods

n/a	Involved in the study
☒	☐ ChIP-seq
☒	☐ Flow cytometry
☒	☐ MRI-based neuroimaging

Antibodies

Antibodies used

human monoclonal anti-Ebola virus Zaire NP, clone KZ51(Absolute antibody, Cat#ab00692-10.0).
mouse monoclonal anti-Ebola virus Zaire VP35, clone 6C5 (Kerafast, Cat#EMS702).
rabbit monoclonal anti-Ebola virus Zaire VP30, clone #1-1 (A kind gift from Dr. Yoshihiro Kawaoka at the University of Wisconsin Madison).
mouse monoclonal anti-Ebola virus Zaire VP24, clone 21-7.7 (A kind gift from Dr. Yoshihiro Kawaoka at the University of Wisconsin Madison).
mouse monoclonal anti-HA tag, clone 16B12 (Biolegend, Cat#901513).
rabbit polyclonal anti-Ebola virus Zaire L (IBT Bioservices, Cat#0301-045).
mouse monoclonal anti-FLAG clone M2 (Sigma-Aldrich, Cat#F1804).
rabbit monoclonal anti-V5 tag, clone D3H8Q (Cell signaling technology, Cat#13202).
mouse monoclonal anti-beta Actin, clone AC-15 (Santa Cruz Biotechnology, Cat#sc-69879).
goat anti-Human IgG (H+L) Cross-Adsorbed Secondary Antibody, Alexa Fluor™ 568 (Invitrogen, Cat#A21090).
goat anti-Mouse IgG (H+L) Highly Cross-Adsorbed Secondary Antibody, Alexa Fluor™ 647 (invitrogen, Cat#A21236).
goat anti-Mouse IgG (H+L) Cross-Adsorbed Secondary Antibody, Alexa Fluor™ 488 (Invitrogen, Cat#A1101).
goat anti-Rabbit IgG (H+L) Cross-Adsorbed Secondary Antibody, Alexa Fluor™ 568 (Invitrogen, Cat#A11011).
Goat anti-Rabbit IgG (H+L) Highly Cross-Adsorbed Secondary Antibody, Alexa Fluor™ Plus 647 (Invitrogen, Cat#32733).

Validation

The mouse monoclonal anti-Ebola virus Zaire VP24 was validated in Nanbo, A., Watanabe, S., Halfmann, P. & Kawaoka, Y. The spatio-temporal distribution dynamics of Ebola virus proteins and RNA in infected cells. Sci Rep 3, 1206, doi:10.1038/srep01206 (2013).
The rabbit monoclonal anti-Ebola virus VP30 was validated in Fang, J., Pietzsch, C., Tsaprailis, G., Crynen, G., Cho, K. F., Ting, A. Y., Bukreyev, A., de la Torre, J. C., & Saphire, E. O. (2022). Functional interactomes of the Ebola virus polymerase identified by proximity proteomics in the context of viral replication. Cell reports, 38(12), 110544. https://doi.org/10.1016/j.celrep.2022.110544.
The usage of the anti-Ebola NP (https://absoluteantibody.com/product/anti-ebola-nucleoprotein-kz51/), anti-Ebola VP35 (https://www.kerafast.com/productgroup/1065/anti-zaire-ebola-virus-vp35-protein-c-terminal-6c5-antibody?ProductID=1008), anti-FLAG M2 (https://www.sigmaaldrich.com/US/en/product/sigma/f1804), anti-V5 (https://www.cellsignal.com/products/primary-antibodies/v5-tag-d3h8q-rabbit-mab/13202) on western-blotting and immunofluorescence analysis, the usage of the anti-Ebola L (https://www.ibtbioservices.com/product/0301-045/), anti-HA (https://www.biolegend.com/en-us/products/anti-ha-11-epitope-tag-antibody-11071?GroupID=GROUP26), and anti-beta Actin (https://www.scbt.com/p/beta-actin-antibody-ac-15) on western-blotting have been provided on the manufacturer's websites.
The usage of the anti-Human secondary antibody, Alexa Fluor 568 (https://www.thermofisher.com/antibody/product/Goat-anti-Human-IgG-H-L-Cross-Adsorbed-Secondary-Antibody-Polyclonal/A-21090), anti-mouse secondary antibody Alexa Fluor 647 (https://www.thermofisher.com/antibody/product/Goat-anti-Mouse-IgG-H-L-Highly-Cross-Adsorbed-Secondary-Antibody-Polyclonal/A-21236), anti-mouse secondary antibody Alexa Fluor 488 (https://www.thermofisher.com/antibody/product/Goat-anti-Mouse-IgG-H-L-Cross-Adsorbed-Secondary-Antibody-Polyclonal/A-11001), anti-Rabbit secondary antibody Alexa Fluor 568 (https://www.thermofisher.com/antibody/product/Goat-anti-Rabbit-IgG-H-L-Cross-Adsorbed-Secondary-Antibody-Polyclonal/A-11011), and anti-Rabbit secondary antibody Alexa Fluor 647 (https://www.thermofisher.com/antibody/product/Goat-anti-Rabbit-IgG-H-L-Highly-Cross-Adsorbed-Secondary-Antibody-Polyclonal/A32733) on immunofluorescence have been provided on the manufacturer's websites.

Eukaryotic cell lines

Policy information about cell lines and Sex and Gender in Research

Cell line source(s)

Human embryonic kidney cells HEK 293T (ATCC reference: CRL-3216).
Vero cells stabling expressing Ebola virus VP30 protein (Vero-VP30) were obtained from Dr. Yoshihiro Kawaoka (University of Wisconsin Madison) as described in Halfmann, P. et al. Generation of biologically contained Ebola viruses. Proc Natl Acad Sci U S A 105, 1129-1133, doi:10.1073/pnas.0708057105 (2008).

Authentication

HEK 293T cells were authenticated by ATCC. Vero cells stabling expressing Ebola virus VP30 protein was not authenticated.

Mycoplasma contamination

Cell lines were not tested for mycoplasma contamination.

Commonly misidentified lines<br>(See ICLAC register)	None.
